# Control of Bovine Viral Diarrhea

**DOI:** 10.3390/pathogens7010029

**Published:** 2018-03-08

**Authors:** Volker Moennig, Paul Becher

**Affiliations:** Institute of Virology, University of Veterinary Medicine, Bünteweg 17, D-30559 Hannover, Germany; Paul.Becher@tiho-hannover.de

**Keywords:** bovine viral diarrhea, viral persistence, systematic control, role of vaccination

## Abstract

Bovine viral diarrhea (BVD) is one of the most important infectious diseases of cattle with respect to animal health and economic impact. Its stealthy nature, prolonged transient infections, and the presence of persistently infected (PI) animals as efficient reservoirs were responsible for its ubiquitous presence in cattle populations worldwide. Whereas it was initially thought that the infection was impossible to control, effective systematic control strategies have emerged over the last 25 years. The common denominators of all successful control programs were systematic control, removal of PI animals, movement controls for infected herds, strict biosecurity, and surveillance. Scandinavian countries, Austria, and Switzerland successfully implemented these control programs without using vaccination. Vaccination as an optional and additional control tool was used by e.g., Germany, Belgium, Ireland, and Scotland. The economic benefits of BVD control programs had been assessed in different studies.

## 1. Introduction

Bovine viral diarrhea (BVD) was discovered in 1946 by researchers at Cornell University [1]. It was described as a new, rather inconspicuous transmissible diarrhea. It became clear that BVD was ubiquitous in cattle populations worldwide, but its impact on animal health was greatly underestimated for a long time. Today, the economic impact of this infection is fully appreciated [2]. Mucosal disease (MD) of cattle was first described in 1953 [3], and it took years until it became clear that MD was also caused by BVD virus (BVDV). Another milestone in our understanding of BVD was the finding that the virus could cross the maternal placenta at any time with varying results. Of special significance was the fetal inability to recognize the invading virus during the first stages of pregnancy. Resulting persistently infected (PI) calves tolerant to their endogenous BVDV proved to be the key for the virus’ success to survive and persist in cattle populations [4]. Based on their effects on cell cultures, two biotypes of BVDV are known, the noncytopathic (ncp) and cytopathic (cp). Only ncp BVDV are able to establish persistent infections, while the emergence of cp BVDV by spontaneous mutation in animals persistently infected with ncp BVDV is crucially implicated in pathogenesis of MD [5,6]. BVDV displays considerable genetic variation and two different species (BVDV-1 and BVDV-2) have been recognized, each comprising several subgenotypes [7,8]. The more that became known on the impact of BVD on cattle health and economy, the greater the necessity for its control became apparent [9,10]. However, since the virus was endemic and all available laboratory tools for the virological and serological diagnosis were based on time- and cost-intensive tissue culture techniques, it was long thought impossible to effectively control the infections and resulting disease conditions.

## 2. Control of BVD Using Vaccination

One of the preferred methods for the control of infectious diseases is vaccination, since it is relatively inexpensive and effective. 

The original purpose of vaccination against BVD was prevention of clinical disease and transient immunosuppression caused by BVDV. Efficacy was not evaluated with respect to fetal protection. Thus, vaccination did not necessarily prevent the emergence of new PI cattle, the virus reservoir was maintained, and infection of susceptible animals continued. When the significance of PI animals for the perpetuation of the infection was recognized, the target of vaccination shifted to the primary goal of fetal protection. 

The first modified live vaccine (MLV) against BVD was developed in the early 1960s [11]. Thereafter, more MLVs were developed and widely used, often in combination with other agents [12]. In general, these vaccines were quite efficacious, however, there have been reports about adverse effects, which were usually attributed to the immunosuppressive effects of live vaccine virus or the intrauterine infection of pregnant animals with either vaccine virus or BVDV field virus contaminating the vaccine. The source of the latter was usually fetal calf serum that was essential to grow cells producing the vaccine virus, and that had the potential to interfere with vaccine production [13,14]. Most modern MLVs are cpBVDV, because cytopathic viruses are not able to establish a persistent infection in the fetus. MLV induce a strong humoral and cellular immunity and they provide a solid fetal protection. In Europe, the use of MLV is not recommended in unvaccinated animals during the first six months of pregnancy. Calves at the age of seven weeks of age can be vaccinated against BVD, and they develop a T-cell mediated immune response despite circulating maternal antibodies [15]. Although different studies are difficult to compare, this effect seems to be age-related. In younger calves (10–14 days), vaccination with BVDV-1 was blocked by high maternal antibodies. The animals were later challenged with BVDV-2 [16]. MLV based on ncpBVDV should not be used in pregnant cattle because they cross the placenta infecting the developing fetus. In order to overcome safety concerns associated with ncpBVDV a mutant virus was developed, where the Npro gene was deleted and the endoribonuclease activity of Erns was inactivated. This ncpBVDV was not able to cross the placenta of pregnant cattle, and it elicited an immune response comparable to a BVDV field isolate [17,18].

Safety concerns have prompted the development of killed vaccines (KVs), which could be applied at any age and stage of pregnancy [19]. In comparison to MLVs, KVs have to be injected several times to achieve protection, and onset of immunity takes at least three to four weeks, whereas MLVs confer protection within a few days. Fetal protection varies from incomplete [20] to satisfactory [19,21]. The immune response to KVs has been improved in recent years by adding powerful adjuvants. Humoral immunity after the application of KVs is usually strong, and the cellular immunity varies from incomplete to strong [22,23]. However, a possible risk associated with KVs with powerful adjuvant formulations became apparent with a BVD vaccine that induced alloantibodies leading to the emergence of bovine “neonatal pancytopenia” (BNP). It is likely that adjuvants not only augmented the immune response against viral antigens but also to cellular debris from bovine cells used for virus production [24,25]. This effect is considered to be of fundamental significance for the development of future KVs [26]. In Europe, there are mainly inactivated BVD vaccines on the market. 

Another strategy in order to overcome safety concerns with MLV is the combination of repeated inoculations of KVs and MLVs or vice versa. In 2002, Frey and coworkers reported a two-step vaccination procedure for the improvement of reproductive protection against BVD: heifers were immunized with KV and four weeks later, boosted with MLV [27,28]. Annual revaccinations were recommended using KV. A similar approach for the improvement of reproductive protection was developed by Walz and coworkers [29]. 

The purpose of any systematic vaccination is to prevent new infections, reduce viral shedding, and increase herd immunity. Only when there is a basic reproduction ratio of R_0_ < 1, i.e., one infected animal infects less than one other susceptible animal, the infection dies out. With most acute infections, the percentage of non-susceptible animals to reach that threshold varies between 50–95% [30]. Vaccination will only be successful when a minimum coverage of the population is achieved with enough non-susceptible animals. There are several animal infections which can be successfully controlled by systematic vaccination. The most striking example was rinderpest, which had been eradicated worldwide by the year 2011 [31], but classical swine fever (CSF) [32], rabies [33], and pseudorabies [34], among other diseases, can also be effectively controlled using vaccination. 

Is vaccination against BVD comparable to these aforementioned viral infections? Despite progress in the development of BVD vaccines, the assessment of the effect of widespread vaccination against BVD over several decades is disappointing. Vaccination has not changed BVD prevalence over time [35]. The reason for this failure is based on the unique biology of BVD infections, which was not fully understood for a long time, and which is still widely underestimated. The ability of BVDV to produce PI offspring creates a virus reservoir that is permanently shedding large amounts of infectious virus. Thus, PI cattle have ample opportunity during their lifetime to meet and infect new susceptible animals, even when the general level of immunity against BVD is high. This is in contrast to most other infectious diseases where shedding is temporally limited to a few days, or rarely, weeks. Even viruses inducing latent infections, e.g., herpesviruses, are not shed all the time, but rather over short periods. In an environment with a high proportion of immune animals, single acutely infected animals will not be able to meet and infect enough remaining susceptible animals in order to maintain or even spread the infection. 

In this respect, BVD cannot be compared with other infectious diseases, because it can be assumed that in the presence of PI cattle, only a population immunity of 100% will prevent the emergence of new PI calves. This was observed in small herds with one or a few PI animals. Constant booster of the rest of the herd led to complete immunity and no fresh fetal infections in pregnant animals. After the death of the last PI animal, the herd was BVD-free (self-clearance) [36,37,38]. However, this effect might be less frequent in larger herds, since a 100% solid immunity is almost impossible to achieve under practical conditions. As a consequence, a successful “vaccination only” strategy for a sustainable BVD control in regions or countries had never been reported.

## 3. Control of BVD without Vaccination

With improvements of diagnostic technologies, namely the development of ELISA techniques for the detection of BVDV antigen and virus-specific antibodies, respectively, cost-effective mass screenings became possible [39,40,41]. Virological screening allowed the identification and elimination of PI animals, thereby depriving BVDV of its reservoir in single herds or in cattle populations. Later, the development of PCR protocols for the identification of BVDV added sensitivity and the option of investigating pooled samples [42]. Serological screening of random serum or bulk milk samples made the assessment of the status of individual animals or herds possible. 

The Scandinavian countries were the first to take advantage of cost-effective diagnostic methods. Since BVD vaccination had never been used in these countries, it was possible to identify herds with an active BVD infection using serological methods. Once random blood or bulk milk samples indicated fresh infections in a herd, a search for PI animals began. Infected animals were removed, and infected herds were under movement restrictions in order to prevent further spread of the virus. The herds remained under surveillance until the virus was eliminated. After having cleared herds from PI, cattle they were monitored regularly. All Scandinavian programs were successful, and after a few years, the countries were largely free from BVDV. Retrospect analyses confirmed a favorable cost benefit of the programs [10]. 

In Sweden, a BVD control program was initiated in 1993. In the first stages, it was voluntary, and later became compulsory on request by the dairy industry [43,44]. During its first two years, the program was funded privately, and thereafter, it was supported by the Swedish government. Similarly, a program was started by the stakeholders in Norway in 1993. During its last two years, the program was controlled by the state veterinary authorities [10]. The Danish program was based on the same principles, and was started in 1994 [45,46]. Finland had an exceptionally favorable situation, with only a very few BVDV-infected herds when starting the control program, which was in place between 1998 and 2004 [47]. Encouraged by the success of the Scandinavian programs, Austria initiated a similar control program in 2004 [48]. 

The Swiss BVD control program started in 2008. Due to the high prevalence of BVD (>80% BVD antibody-positive cattle) in the national cattle population, the strategy differed from the Scandinavian approach: in the first year, all Swiss cattle were investigated virologically, and PI cattle were removed. During the following four years (2009–2012), all newborn calves were tested for BVDV using ear-notch samples. These measures reduced the PI prevalence from 1.3% to 0.02% [49]. Since 2013, a serological surveillance scheme is in place. Dairy herds are monitored using bulk milk samples and groups of animals in non-dairy herds are monitored by blood serology. As in Scandinavia and Austria, vaccination against BVD was banned. 

When controlling BVD without vaccination, the final phases of the program present one major problem: in cattle populations with endemic BVD, the seroprevalence is usually high and may exceed 90% of the animals. New infections of herds with BVDV have no dramatic consequences, since there are relatively few susceptible cattle. With the systematic removal of PI animals, the infectious pressure is greatly reduced, and the cattle population becomes seronegative. Reintroduction of BVDV into susceptible herds results in maximum damage with respect to acute clinical disease and effects on fertility. This effect has been reported from almost all countries with systematic control programs without vaccination. In Scandinavia, it has been described by several authors [38,46], and in Switzerland, the number of newly recognized PI calves increased in the first years after 2012, because the traditional grazing on alpine pastures where cattle from multiple herds mix facilitated BVDV reinfections. As a consequence, epidemiological investigations implementing the national cattle database were intensified [50]. Despite setbacks, the situation is considered to be stable, and a favorable cost benefit analysis was reported [51]. In Austria, currently, the vast majority of cattle herds are certified free from BVD with only a few infected herds remaining. Molecular analysis is being used to trace new outbreaks [52].

The vulnerability of susceptible herds to reinfection underlines the necessity of strict biosecurity as an integral part of any BVD control program. 

## 4. Combined Control Programs

Any successful BVD control program requires the removal of PI animals in order to protect susceptible or incompletely protected animals. Based on this hypothesis, the first voluntary control program in Germany was initiated in the federal state of Lower Saxony in the early 1980s [53]. The aim of the effort was to clear infected herds from PI cattle. Immediately after detection, viremic cattle had to be removed from the herd, and they were partly compensated by a parastatal insurance. The voluntary nature of the program turned out to be a critical disadvantage of this policy: after the removal of all PI animals, participating herds became seronegative, and hence, fully susceptible for new infections. Since the program was not compulsory, infective pressure from neighboring herds not taking part in the program was still high, and reinfections with severe economic consequences were frequent. The policy had to be amended, and herds had to be vaccinated after clearing of PI cattle, in order to prevent accidental reinfection [54]. However, due to the voluntary approach, only participating herds benefitted from the program and progress was slow. On the national level, a federal guideline for the control of BVD was issued; in 2004 BVD became notifiable, and in 2011 a national control program started. Since Germany has several cattle-dense regions with about 200 animals per km^2^ and intense trade, a decision was made to follow the combined approach of test and cull plus optional vaccination. Newborn calves were tested for BVDV using ear-notch samples. Positive animals are being retested after at least 40 days, in order to rule out transient infection. After identification, PI animals have to be culled. Depending on the local epidemiological situation, vaccination can be banned or ordered by the veterinary authorities. In practice, however, the decision whether to vaccinate or not is often left to the veterinary practitioner and the farmer. So far, the program has been quite successful, and the PI prevalence dropped from 0.48% in 2011, to 0.02% in 2016. The exit strategy has not yet been decided, i.e., when to stop collection of ear-notch samples from calves and how to monitor the situation afterwards [55]. 

There are at least three more countries using a combined approach to control BVD: Scotland, Ireland, and Belgium. The Scottish BVD control scheme started in 2010, and comprised different stages, ranging from subsidized screening to enhanced testing and movement restrictions. Vaccination may be used where appropriate [56]. 

After a voluntary phase, the compulsory Irish program started in 2013. All newborn calves (including stillbirths) had to be tested by ear-notch sample in designated laboratories. Vaccination is allowed as a control tool [57]. The prevalence of PI cattle has decreased from 0.66% in 2013 to 0.1% in 2017 [58].

## 5. Genetic Diversity of BVDV and Related Viruses and Its Impact on Control of BVD

BVD control programs have to take into account the genetic variability of pestiviruses. The broad host spectrum of BVDV might also interfere with control measures.

BVDV is the type species of the genus *Pestivirus* within the *Flaviviridae* family, which also comprises the genera *Flavivirus*, *Hepacivirus*, and *Pegivirus* [59]. Molecular characterization of BVDV strains in the 1980s and early 1990s resulted in the establishment of the first viral genomic sequences providing the basis for the segregation of BVDV strains into two separate species, BVDV-1 and BVDV-2, which can be further subdivided into subgenotypes [7,60,61,62,63]. Similar to hepaciviruses and pegiviruses, analysis of a growing number of pestivirus strains revealed an expanding genetic diversity and host range [8,64,65,66,67,68,69,70,71,72,73,74]. Considering the continuously growing number of tentative virus species, the taxonomy of the *Flaviviridae* has been recently updated, and a revision of the nomenclature of hepaci-, pegi-, and pestiviruses has been proposed by the *Flaviviridae* Study Group of the *International Committee on Taxonomy of Viruses* [59,75,76]. According to this proposal, pestivirus species are named in a host-independent manner using the format *Pestivirus X*. Only the virus species names are changed, and virus isolates are still referred to by their original names [76]. BVDV-1 and BVDV-2 are represented by the species *Pestivirus A* and *Pestivirus B.*

Similar to other plus-strand RNA viruses, an extensive genetic variability can be observed for BVDV-1, BVDV-2, and other pestiviruses. In addition to accumulating point mutations due to the lack of proofreading activity of the viral RNA-dependent RNA polymerase implicated in viral genome replication, replicative and non-replicative RNA recombination contribute to pestivirus evolution [77,78,79,80]. To date, partial genomic sequences have been reported for thousands of BVDV-1 and BVDV-2 isolates, while information for the other pestiviruses infecting cattle is limited [80]. For BVDV-1, at least twenty-one subgenotypes (BVDV-1a-1u) have been described, while BVDV-2 can be segregated into four subgenotypes (BVDV-2a-2d) [80]. Our current knowledge on the global distribution of BVDV subgenotypes and their occurrence in individual countries has been recently summarized [80]. Both virus species were detected across all of the inhabited continents with an overall significantly higher prevalence for BVDV-1. BVDV-1a to 1c, and BVDV-2a were most frequently reported. In different countries, various collections of BVDV subgenotypes have been described. The occurrence of variations at nucleic acid level can have direct consequences for BVDV diagnosis, as mutations at the primer target sites in the viral genome may prevent detection of such BVDV variants by established RT-PCR assays. The failure of detection of BVDV genomes may severely interfere with control programs depending on reliable detection and subsequent elimination of PI animals. A detailed knowledge of the genetic diversity of BVDV and related viruses can provide useful insights into the genetic relatedness among these viruses, which are either endemically present in an area for a longer time period or have been recently introduced e.g., by animal imports. Accordingly, studies on molecular epidemiology of BVDV can assist in tracing virus isolates circulating in individual countries and provide useful information for evaluating the success of disease control programs [50,80,81,82,83,84,85,86,87,88,89,90,91,92]. 

BVDV can infect a wide range of animals belonging to the order *Artyodactyla*, including cattle, sheep, goat, other domestic and wild ruminant species, old and new world Camelidae, as well as domestic pigs and wild boar [63,64,80,93,94]. Apart from cattle, BVDV-1 and BVDV-2 have been frequently detected in sheep, and in some countries BVDV infections in sheep are more abundant than infections with BDV [64,95]. A high prevalence of BVDV-1 and BVDV-2 in sheep and the ease of virus transmission from sheep to cattle should be considered as a potential threat to the success of BVD control and eradication programs. While transmission of BVDV from wild ruminants to cattle can sporadically occur, there is so far no evidence that wild animal species have significantly interfered with BVD control and eradication programs in Europe. The situation in North America is less clear, because PI white-tailed deer are considered to be a low risk reservoir for the cattle population [96,97]. 

In addition to BVDV-1 and BVDV-2, other ruminant pestiviruses, including Border disease virus (BDV; *Pestivirus D*), giraffe pestivirus-like viruses (*Pestivirus G*), and Hobi-like viruses, also known as BVDV-3 (*Pestivirus H*), are able to infect cattle under natural conditions [64,72,98,99,100,101,102]. While infections of bovine with BDV are very rare, in some regions like in Northeastern Brazil, Hobi-like pestiviruses have been more frequently detected in cattle than BVDV-1 and BVDV-2 [103]. It is well known that BVDV-1, BVDV-2, BDV, and other ruminant pestiviruses are antigenically related, and antibodies induced after infection with a given pestivirus (e.g., BDV) can cross react with antigens of heterologous pestivirus species. Accordingly, the antigenic similarity of BVDV, BDV, and other ruminant pestiviruses may become a problem for demonstrating freedom of BVD by serology in the cattle population [100].

Antigenic relationships among pestivirus species have been analyzed by using monoclonal antibodies and by cross-neutralization studies. These studies demonstrated significant differences between members of different species, and to a lower extent, even between different BVDV-1 subgenotypes [62,70,98,104,105,106,107]. While it has been shown that a BVDV-1 vaccine can induce cross-protection against a BVDV-2 challenge [108], different levels of cross-protection towards highly variable BVDV-1 and BVDV-2 subgenotypes might be implicated in the success of vaccination programs. 

## 6. Conclusions

Several studies on the economics of BVD control clearly show that the cost benefit ratio of systematic BVD control is favorable [109]. However, implementation is sometimes difficult, due to a lack of sound control programs. Compulsory and systematic control programs have been shown to be most successful. In several European countries, the test and cull strategy without vaccination has yielded convincing positive results with a favorable cost benefit ratio. In difficult circumstances, e.g., high cattle densities, intense trade, and high seroprevalence against BVD, the test and cull strategy might be supplemented by vaccination, in order to protect susceptible cattle. This approach was also shown to be successful. However, all control programs, and in particular, all vaccination approaches have to take the genetic diversity of BVDV into account. All control efforts have to be accompanied by strict biosecurity and—where appropriate—movement restrictions. Voluntary programs may be successful in their initial phase but expensive in the long run, and risk of ultimate failure is high. A vaccination only strategy has never been shown to be suitable for sustained success.
